# Connecting digital food literacy and sustainable eating in children and adolescents: a focused mini review of evidence gaps and educational priorities

**DOI:** 10.3389/fnut.2026.1953673

**Published:** 2026-09-22

**Authors:** Lu Xing, Xueqi Wu

**Affiliations:** 1 School of Preschool Education, Changsha Normal University, Changsha, China; 2 School of Education, Faculty of Social Sciences & Leisure Management, Taylor’s University, Subang Jaya, Malaysia; 3 Taylor’s Culinary Institute, Faculty of Social Sciences & Leisure Management, Taylor’s University, Subang Jaya, Malaysia

**Keywords:** adolescents, children, digital food literacy, digital interventions, food education, food systems, food waste, sustainable diets

## Abstract

Children and adolescents learn about food through digital and physical environments while developing an understanding of dietary effects on human and planetary health. Digital healthy-eating education, food-literacy education, and sustainable-diet education have nevertheless developed along largely separate trajectories. This focused Mini Review examines interventions that combine a substantive digital educational mechanism, a food-literacy capacity, and an explicit sustainability target in young people. Database-specific searches of Web of Science, Scopus, PubMed, and ERIC plus citation checking yielded three eligible studies; exploratory high-sensitivity checks did not reveal an evident additional match. The studies evaluated an adolescent food-systems simulation with guided inquiry, online family cooking with text-message support, and a seven-mission family mHealth program addressing food waste and plant-based proteins. They provide preliminary evidence for three educational configurations rather than a basis for comparative effectiveness claims. Mixed Methods Appraisal Tool assessment identified credible alignment between questions and data, alongside immediate qualitative outcomes, single-arm designs, selective outcome completion, and limited control of confounding. The synthesis distinguishes food literacy delivered through digital media—the eligibility construct—from critical literacy about digital food environments, which remains a proposed research direction. Current evidence supports explicit specification of digital affordance, literacy capacity, sustainability target, participant role, outcome tier, and inferential strength. Future trials should recruit more diverse populations, strengthen child-led participation, separate child and household outcomes, and test longer-term behavioral and material effects.

## 1 Introduction

Food learning now occurs across kitchens, classrooms, school meals, games, websites, social media, and commercial platforms. For children and adolescents, the boundary between online and offline food environments is increasingly porous. Digital tools can simulate food systems, provide repeated prompts, connect educators with families, and make culturally adapted resources accessible. The same environment can amplify commercial marketing, misinformation, algorithmic personalization, and unequal exposure to food-related content. Reviews of the digital food environment therefore emphasize that technology is not a neutral channel: platform architecture and commercial practices shape what young people encounter and how food choices are framed (1, 2).

Food education is also moving beyond nutrient knowledge and individual choice. Sustainable eating requires attention to food production, waste, environmental impact, cultural meaning, access, and the institutional settings in which choices become possible. Ares et al. (3) describe children’s food literacy through relational, functional, and critical capacities. Relational literacy concerns meanings, values, identity, and relationships around food; functional literacy includes knowledge and practical skills; and critical literacy enables young people to examine claims, systems, power, and trade-offs. This wider framing connects healthy eating with food waste, production systems, dietary carbon, labor, affordability, and competing environmental claims.

Three substantial, partly overlapping literatures have emerged. Technology-enabled adolescent food-literacy programs were synthesized by Wickham and Carbone (4), while Prowse and Carsley’s (5) umbrella review and Talens et al.’s (6) review show that digital interventions for young people commonly target healthy eating, obesity prevention, and related behaviors. Two recent all-age reviews moved closer to the sustainability intersection: Vanwinkelen et al. (7) synthesized 52 digital intervention studies focused mainly on plant-based eating or meat reduction, and Gardini et al. (8) reviewed 16 studies using psychological and behavioral strategies for healthy and sustainable food choice. Both reported limited developmental analysis. In parallel, school-based reviews have examined elementary food literacy, primary-school sustainable-diet programs, and adolescent perceptions and interventions (9–11). These bodies establish the need for a child-focused synthesis that treats digital pedagogy, food-literacy capacity, and explicit sustainability as simultaneous requirements.

This review uses digital food literacy in two related but distinct senses. The eligibility construct is food literacy developed through digital media: a digital component must substantively deliver, mediate, personalize, simulate, or structure learning. A second meaning—critical literacy about digital food environments, including influencer claims, targeted marketing, algorithms, and misinformation—is treated as wider-literature-informed future work unless an intervention explicitly teaches and evaluates it. Keeping these meanings separate also distinguishes digital pedagogy from digital delivery, measurement, and exposure.

The distinction is consequential for intervention design. A website that stores worksheets, a camera used to measure plate waste, an algorithm that tailors feedback, and a simulation that represents a food supply chain all involve technology, but they do not perform the same educational work. Describing only the platform can conceal the active learning process and frustrate replication. The useful unit of comparison is therefore the affordance–activity pair: what the technology allows a learner or family to do, and how that activity is expected to change a defined literacy capacity. Sustainability then requires its own specification because improved dietary quality, reduced waste, lower-impact substitution, and food-systems reasoning are related but non-equivalent outcomes.

The review asks: (1) what empirical evidence combines digital food-literacy education with explicit sustainable-eating targets in children and adolescents; (2) which boundaries distinguish that intersection from digital healthy-eating education, digital food environments, and sustainability-oriented food education; and (3) which educational and evaluation priorities follow from the included evidence and the wider literature?

## 2 Focused review approach

Web of Science Core Collection, Scopus, PubMed, and ERIC were searched on 30 July 2026 for English-language peer-reviewed intervention evaluations published from 2000 through the search date. Database-specific strings combined population terms; digital media and food/nutrition literacy or education; and sustainability terms including sustainable diets, food systems, food waste, plant-based eating, environmental sustainability, and responsible consumption. The original searches yielded 53 records and 38 unique database candidates after DOI and normalized-title deduplication. The sole supplementary candidate—Eckert et al.’s (12) published Supper Heroes report—was identified in the included-study table of Vanwinkelen et al. (7) and met the same criteria, giving 39 records for screening. Full reproducible strings, the search log, and the supplementary-candidate provenance are provided in Appendix S1 and Supplementary Table S1.

On 20 August 2026, exploratory high-sensitivity searches added internet-based, mHealth, eHealth, text-message, multimedia, video, serious-game, simulation, remote, plant-forward, meat-reduction, food-loss, and carbon-footprint terms. Raw, non-additive counts were 154 in Web of Science, 184 in Scopus, 96 in PubMed, and 35 in ERIC. Title and metadata inspection in Scopus, PubMed, and ERIC, supplemented by Web of Science interface checks, identified no evident additional eligible report among the records examined. These checks tested vocabulary sensitivity; no updated cross-database deduplicated corpus or second formal study flow was constructed.

Direct inclusion required: participants aged 5–18 years (P1); a substantive digital educational mechanism (D1); at least one relational, functional, or critical food-literacy capacity (L1); an explicit sustainability target in content or outcomes (S1); and a published intervention evaluation (I1). Digital measurement alone, observational exposure, health outcomes without an environmental or food-systems dimension, protocols, and non-digital education failed the direct criteria. Twelve records required full-text assessment: three met all five criteria; five lacked substantive digital pedagogy; three were observational, conceptual, or protocol reports rather than intervention evaluations; and one lacked an explicit sustainability target in evaluated content or outcomes. Backward reference lists of the three included reports and forward citations available at the update were checked. Boundary records originated in the same search and were assigned to prespecified, mutually exclusive adjacent or contextual categories. Supplementary Table S2 gives the decision, full-text status, failed gate, and reason for every screened record.

Study characteristics were coded by digital affordance, literacy capacity, sustainability target, developmental and implementation context, participant level, and outcome tier. T1 denotes a direct material or environmental measure, T2 a measured sustainable behavior, T3 knowledge, attitudes, intentions, or immediate learning, and T0 no explicit sustainability outcome. Methodological quality was appraised with the 2018 Mixed Methods Appraisal Tool (MMAT), using design-specific criteria and item-level judgments rather than a summary score (13). No study was excluded on quality. One reviewer conducted screening, coding, and appraisal; Supplementary Tables S2, S3 report the record-level decisions and item-level judgments. Supplementary Figure S1 presents the study-selection flow; Figure 1 maps the eligibility and evidence architecture; and Table 1 compares the three direct studies.

**Figure 1:**
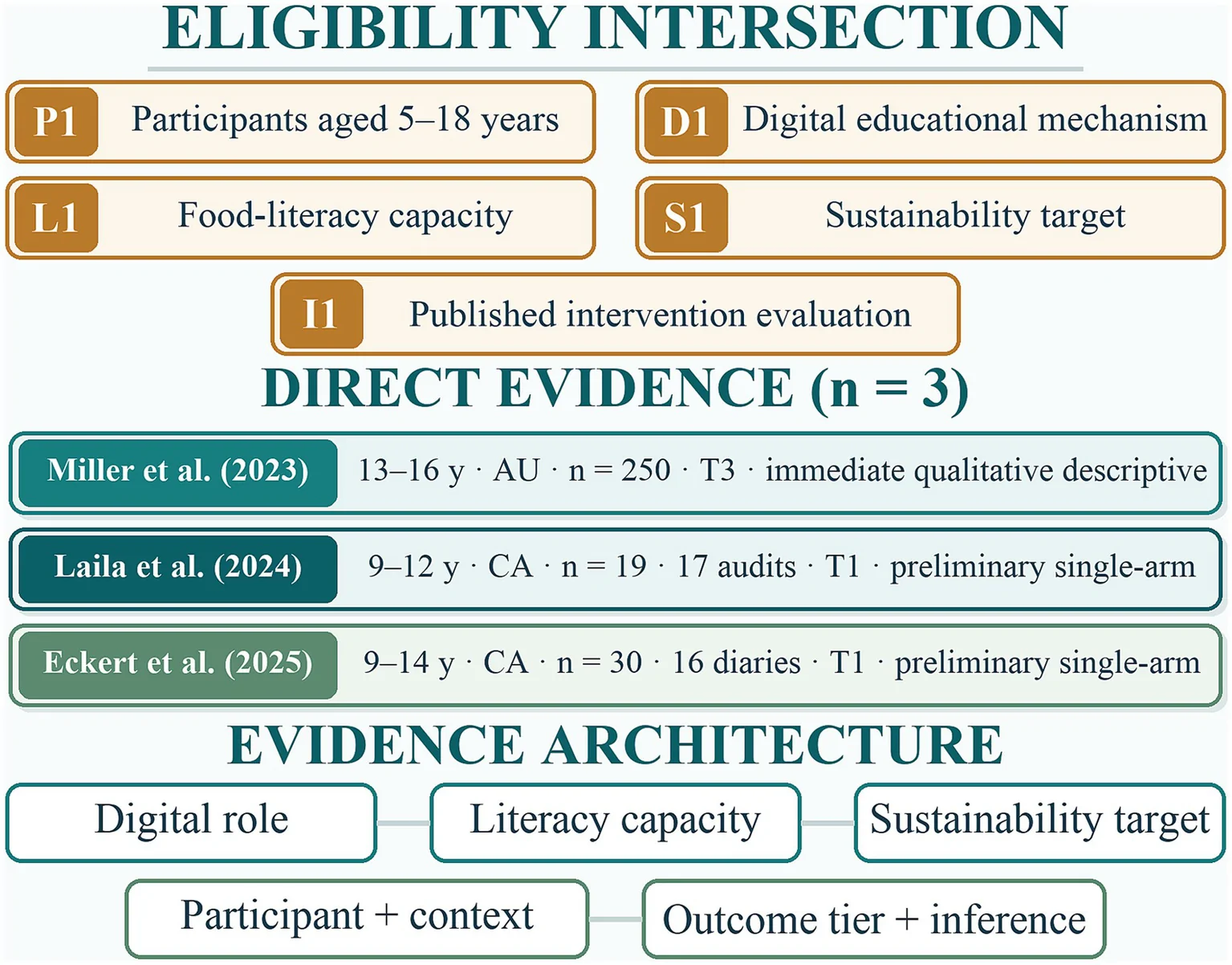
Evidence architecture for the direct intersection.

**Table 1:** Direct evidence connecting digital food-literacy education with explicit sustainable-eating targets.

Study and design	Population, age, setting, and analysis denominator	Digital educational mechanism	Sustainability outcomes	Evidence role and inferential strength
Miller et al. (14); qualitative case study	250 students; aged 13–16 years; five secondary schools; Australia; group-discussion records thematically analyzed	Food-systems simulation followed by facilitated inquiry; systems-oriented knowledge and discussion	Production, resource and food waste, and human/planetary health; T3 immediate group-discussion outcomes	Immediate qualitative descriptive evidence; coherent qualitative design; no behavioral follow-up
Laila et al. (15); single-arm pre–post pilot	19 families at baseline; 18 attended; 17 mothers, 12 fathers, and 21 children completed surveys; children aged 9–12 years; 17 paired household audits; Canada	Online cooking class, toolkit, and four text messages/week; planning, preparation, storage, and leftover skills	T1 household audits: avoidable fruit-and-vegetable and unavoidable other waste decreased; total waste did not change significantly	Preliminary within-group outcome evidence; small convenience sample; single-arm design without a control group
Eckert et al. (12); single-arm pre–post mixed-methods study	30 families at baseline; 25 began; 23 retained; 19 completed all missions; index children aged 9–14 years; 16 paired weighed diaries; Canada	Seven mHealth missions with videos, games, cooking, recipes, and family activities; sustainable-diet knowledge and routines	Food waste and plant-based proteins; T1 paired diaries in 16 families; child best-before-date knowledge improved	Preliminary within-group mixed-methods evidence; single-arm design; selective diary completion; varied household, parent, and child outcomes

Evidence-role labels synthesize study design, outcome denominator, follow-up, and item-level MMAT appraisal; no numerical quality score was assigned. T1 = direct material or environmental outcome; T3 = knowledge, attitudes, intentions, or immediate learning. AU, Australia; CA, Canada.

## 3 Evidence architecture: outcome proximity and inferential strength

In this review, evidence architecture maps the alignment among the digital educational mechanism, food-literacy capacity, sustainability target, participant level, outcome tier, and appraisal-based inferential strength. It serves as a descriptive organizing matrix across these dimensions. Outcome tier and inferential strength are separate: T1–T3 locate outcomes along a learning-to-material continuum, whereas study design and MMAT judgments determine the warranted inference.

### 3.1 Three studies at the direct intersection

Miller et al. (14) evaluated a purpose-built food-systems computer simulation with 250 students aged 13–16 years in five Australian secondary schools. After game play, facilitated group discussions elicited learning and interests. Students discussed crop growth, production, resource and food waste, healthier choices, and links among food systems, human health, and planetary health. The simulation made supply-chain relationships open to manipulation, while discussion required students to articulate observations and propose experiential extensions.

This qualitative case study illustrates simulation-supported systems inquiry. Its immediate group-discussion outcomes sit at T3 and do not establish sustained learning or behavior change. The educational contribution of the simulation also cannot be separated from facilitation.

Laila et al. (15) piloted Weeknight Supper Savers with families of children aged 9–12 years. The four-week program combined a chef-led online cooking class, a food-waste toolkit, and four text messages per week about planning, preparation, storage, and leftover use. Nineteen families completed baseline assessment and 18 attended the cooking class. All 17 mothers and 12 fathers who completed surveys reported satisfaction; among 21 child respondents, 73% were satisfied with the overall program. Paired household audits from 17 families supplied a T1 outcome: avoidable fruit-and-vegetable waste and unavoidable other waste decreased, whereas total food waste did not change significantly.

The program connects synchronous practice and repeated prompts with household routines distributed across shopping, storage, preparation, serving, and leftovers. Its small single-arm design evaluates the package as a whole and provides feasibility and preliminary outcome evidence rather than an estimate of comparative effectiveness.

Eckert et al. (12) evaluated Supper Heroes, a four-month mHealth intervention for families with children aged 9–14 years. Seven online missions combined infographics, videos, matching games, cooking classes, recipes, and family activities to reduce food waste and increase plant-based protein intake. Thirty families completed baseline assessment, 25 began the intervention, 23 were retained to post-intervention assessment, and 19 completed all seven missions.

Among 16 families providing paired weighed diaries, avoidable and possibly avoidable food waste decreased by 1,133 g per week (*p* = 0.002). Mothers increased plant-based protein frequency, fathers did not show a significant change, and children improved recognition of best-before-date meaning while other child food-literacy measures remained stable. The single-arm design, selective diary completion, and highly educated sample limit causal and population inference. The findings provide preliminary evidence of feasibility, acceptability, and possible household change.

Together, the studies describe three configurations: simulation with guided inquiry for adolescents; synchronous online family practice with repeated prompts for preadolescents; and a sustained multimedia family sequence linking food waste, plant-based foods, and child learning. They involve participants aged approximately 9–16 years; none directly evaluates children aged 5–8 years.

Miller therefore contributes immediate qualitative descriptive evidence, whereas Laila and Eckert provide preliminary within-group outcome evidence. These evidence roles reflect design, outcome denominator, follow-up, and item-level appraisal.

The configurations also differ in temporal structure and locus of action. Miller et al. (14) capture learning immediately after a bounded school activity, whereas the family programs distribute learning across meals and household routines (12, 15). In the latter studies, adults control many purchasing, storage, and preparation decisions even when children are named participants. A digital intervention can therefore change child knowledge, adult practice, or a household material outcome without equivalent change at the other levels. This pattern is visible in the mixed findings for Supper Heroes and prevents a single household indicator from being treated as a proxy for child food literacy.

### 3.2 Boundaries of the direct evidence

Four boundaries clarify the three-study denominator. First, digital measurement is not digital pedagogy. Healthy Planet, Healthy Youth used digital photography to quantify plate waste, whereas student-led lessons and school promotion delivered the education (16). Second, digital exposure is not an educational intervention. Seven Avuk et al. (17) linked adolescents’ social-media use with food-choice motives and dietary carbon footprints, but did not deliver literacy education. Third, game structure is not necessarily digital: Wang et al.’s (18) alternate-reality game taught nutrition and reduced measured lunch waste while explicitly operating without digital devices. Fourth, healthy-eating outcomes do not by themselves establish sustainability. Digital child-nutrition reviews offer extensive evidence on tailoring, feedback, and engagement, but the direct intersection requires an explicit construct such as waste, environmental impact, food systems, or a defined lower-impact dietary behavior (5, 6).

Delivery history also matters. FEAST combined food preparation, nutrition, and sustainability, but its online delivery arose partly from pandemic disruption rather than a designed digital mechanism (19). Superhero Foods used online access to extend culturally adapted food-literacy resources to Aboriginal and remote schools, while its evaluated outcomes addressed healthy eating and cultural relevance rather than sustainability (20). These records inform transfer without entering the direct denominator.

The boundary evidence reveals a measurement gradient from immediate learning to material environmental change. Systems-language in a discussion group, a knowledge item about date labels, observed food choice, self-reported plant-protein intake, weighed household waste, and modeled dietary carbon each register a different construct. The T1–T3 labels preserve outcome type rather than rank study quality; outcome proximity and MMAT-based inferential strength are therefore reported separately.

### 3.3 Methodological quality and transferability

MMAT appraisal showed that the Miller study used a coherent qualitative case-study approach, but outcomes were immediate, game-specific, and not linked to subsequent behavior. The two family studies aligned their measures with feasibility and household targets, yet both were small single-arm evaluations without control of secular change or confounding. Weeknight Supper Savers had a convenience sample and reported the multicomponent package as one exposure. Supper Heroes retained 23 of 30 baseline families, but only 16 supplied paired diaries; quantitative inference is therefore especially sensitive to incomplete outcome data. Its qualitative interviews enriched acceptability findings but did not resolve the quantitative design limitations. Item-level judgments appear in Supplementary Table S3.

Geographical and socioeconomic transfer is also limited. All three studies were conducted in Australia or Canada, and the family evidence included comparatively advantaged or highly educated participants. No direct study tested low-resource settings, language adaptations, disability access, or unequal connectivity. The evidence therefore supports design hypotheses and feasibility lessons, not generalized claims across the full 5–18-year age range or diverse food environments.

## 4 Discussion

### 4.1 What the included evidence supports

The direct evidence supports a narrow conclusion: digital components can be embedded in sustainable-food learning through systems simulation, synchronous household skill practice, repeated prompts, and sequenced multimedia activities. Each study names a learning process and an explicit sustainability target. Across studies, heterogeneity, single-arm designs, immediate outcomes, and limited follow-up locate the evidence at the level of preliminary configurations and mechanism-informed hypotheses.

The studies also show why participant level must be explicit. A child can gain systems knowledge, a parent can change purchasing or preparation, and a household can reduce waste. These outcomes occur at linked but different levels. Eckert et al. (12) demonstrate the interpretive risk: household waste, maternal intake, paternal intake, and child knowledge did not change uniformly. This pattern supports separate reporting of who accessed the platform, completed activities, controlled food decisions, and contributed each outcome.

An evidence-aligned design chain follows: specify what the digital component enables; identify the literacy capacity it develops; define the sustainability construct; locate the participant and setting that can act; and select an outcome tier and follow-up interval. Process indicators such as task completion, prompt response, discussion quality, skill rehearsal, and child versus parent participation can test whether the proposed mechanism operated before distal outcomes are interpreted.

The three configurations suggest different mechanisms that require different tests. Simulation externalizes relationships that are difficult to observe directly and can be evaluated through systems mapping, explanation, or transfer to unfamiliar cases. Synchronous cooking coordinates action at a particular meal and can be evaluated through observed skills, confidence, and subsequent independent practice. Repeated messages and multimedia missions distribute cues and activities over time; their contribution requires timestamped exposure and response data rather than a single post-program satisfaction rating. These mechanisms may be complementary, but the current studies do not isolate them. Explicit component descriptions and comparison conditions would allow future reviews to synthesize educational function instead of grouping interventions by device alone.

### 4.2 Wider-literature-informed priorities

The following priorities extend beyond the three included evaluations and should be read as proposals. All-age reviews identify prompts, awareness, decision-making, self-monitoring, self-efficacy, and social interaction as mechanisms worth developmental testing (7, 8). Simulation may support adolescent systems reasoning; synchronous cooking can coordinate household practice; and prompts can extend practice between sessions. Comparative or factorial designs can test these components when sample size permits.

Critical literacy about digital food environments is a distinct future direction. The included studies used technology to deliver or structure learning; none evaluated children’s ability to assess influencer claims, targeted marketing, algorithmic curation, commercial interests, misinformation, or environmental claims. Kraak (21) identifies these capabilities as increasingly important, while reviews document commercial shaping of children’s digital food exposure (2). Future programs can connect learning through digital media with learning about platforms without treating the two constructs as interchangeable.

Developmental fit requires more than simplified language. Younger children may learn through visible routines, stories, games, cooking, and shared family activity, whereas adolescents can deliberate about food-system trade-offs, commercial persuasion, and platform content (3). Because direct evidence begins at about age nine, designs for younger children remain a hypothesis to test. Family participation should support rather than substitute for child agency through age-specific interfaces, collaborative missions, selectable difficulty, and separate reporting of adult and child engagement.

Equity should be built into intervention architecture. Culture, affordability, language, disability, connectivity, device access, and data costs affect both participation and the feasibility of sustainable choices. Culturally relevant characters, foods, narratives, offline alternatives, and shared-device options can support reach (20). Co-design with communities can align sustainability messages with local food availability, cultural meaning, and household constraints.

A school–home sequence offers one way to distribute responsibility without locating sustainability entirely in the child. Classroom simulation or deliberation can make production, waste, and trade-offs visible; a short digital family activity can translate one concept into shopping, storage, cooking, or leftover practice; and a later school discussion can compare constraints and unintended effects. Evaluation can then separate classroom learning, child participation at home, adult action, and household outcomes. Such a sequence also makes structural barriers discussable: limited food access, school procurement, family time, and prices become part of the learning process rather than unexplained nonadherence.

Evaluation should follow the learning objective and sphere of control. Food-waste programs can measure household or plate waste; lower-impact diet programs can assess specified substitutions and defensible environmental indicators; and food-systems programs can evaluate systems reasoning. A planned sequence from learning to practice and material change can reveal where an intervention gains or loses momentum. Longer follow-up, mixed methods, implementation fidelity, and reporting of null or adverse effects—including disengagement, guilt, family conflict, screen burden, and inequitable access—would strengthen cumulative evidence. A school–home model linking classroom inquiry with family practice is one testable configuration, not a conclusion from the current studies.

Measurement development should include a concise common core and a context-specific layer. The common core can report digital exposure, participant identity, literacy capacity, sustainability target, implementation fidelity, outcome tier, and follow-up. Context-specific measures can capture culturally meaningful foods, local waste practices, platform experiences, or control over household decisions. Existing food-literacy instruments vary in age coverage and domain representation, so construct mapping should precede scale selection. Reporting denominators at every stage—eligible, enrolled, exposed, retained, and analyzed—will also prevent participation loss from being hidden behind a single sample size.

### 4.3 Limitations

The review was limited to English-language indexed publications and a narrow conceptual intersection. Terminology remains inconsistent, and citation checking was necessary to recover Supper Heroes. Exploratory sensitivity checks broadened digital and sustainability vocabulary but did not constitute a new deduplicated screening flow; locally indexed or differently labeled studies may remain absent. Boundary studies were systematically categorized but were not synthesized as a second intervention corpus.

Only three heterogeneous studies met all criteria. Their participants were approximately 9–16 years old and located in Australia or Canada; younger children and diverse socioeconomic, cultural, and geographical settings are underrepresented. Formal MMAT appraisal makes study-level limitations explicit but does not create certainty from sparse evidence. Screening, coding, and appraisal were completed by one reviewer; the item-level audit trail improves transparency but does not replace independent verification. These constraints keep conclusions at the level of preliminary configurations, gaps, and testable priorities.

## 5 Conclusion

Three studies provide preliminary models for connecting digital food-literacy education with sustainable eating in young people: food-systems simulation with inquiry, online family cooking with repeated waste-reduction support, and multimedia family learning addressing food waste and plant-based proteins. The evidence supports careful alignment among digital affordance, literacy capacity, sustainability target, participant level, outcome tier, and inferential strength. Comparative effectiveness and generalizability across childhood remain open questions. Clear construct definitions, independent and more diverse evaluations, child-specific outcomes, and longer follow-up are the immediate research needs. Critical literacy about digital food environments is a complementary future agenda that should be explicitly taught and evaluated rather than inferred from digital delivery.
