# Supplementary material for: Connecting digital food literacy and sustainable eating in children and adolescents: a focused mini review of evidence gaps and educational priorities
Source: Front Nutr. 2026 Sep 22;13:1953673. doi: 10.3389/fnut.2026.1953673 (PMC13639947; doi:10.3389/fnut.2026.1953673)
Supplement: Supplementary file 1 [file Supplementary_File_1.docx]

Supplementary Material: Tables and Methods

Table S1. Search and sensitivity-check summary

| **Source** | **Original search date** | **Original raw count** | **Expanded date** | **Expanded raw count** | **Role** |
| --- | --- | --- | --- | --- | --- |
| Web of Science Core Collection | 30 Jul 2026 | 18 | 20 Aug 2026 | 154 | Original flow; exploratory vocabulary check |
| Scopus | 30 Jul 2026 | 15 | 20 Aug 2026 | 184 | Original flow; exploratory vocabulary check |
| PubMed | 30 Jul 2026 | 15 | 20 Aug 2026 | 96 | Original flow; exploratory vocabulary check |
| ERIC | 30 Jul 2026 | 5 | 20 Aug 2026 | 35 | Original flow; exploratory vocabulary check |
| Citation checking | 30 Jul 2026 | 1 additional candidate | 20 Aug 2026 | No new eligible study | Backward/forward and review-list checks |

Note. The four original database exports contained 53 records and 38 unique database candidates. One supplementary candidate produced 39 screened records. Expanded counts are non-additive exploratory checks; no updated deduplicated corpus or second formal flow was constructed.

Supplementary candidate provenance. C039 (Eckert et al., 2025; doi: <https://doi.org/10.1016/j.appet.2025.107849)> was the sole candidate added outside the original database exports. It was identified in the included-study table of Vanwinkelen et al. (2026), assessed against the same P1–I1 criteria, and included as the Supper Heroes study.

# 1 Appendix S1A. Original database-specific search strategies

## 1.1 Reproducible search strategy

## 1.2 Search date and purpose

All formal searches were run on 30 July 2026. The strict branch was designed to locate published records at the intersection of four concepts: (1) a substantive digital medium, (2) food/nutrition literacy or education, (3) an explicit sustainability construct, and (4) children or adolescents. Two broader branches were used only to calibrate field size: digital food/nutrition education in young people, and sustainability-oriented food education in young people. The calibration counts are not screened corpus sizes.

## 1.3 Shared concept blocks

Population (P)

(child* OR adolescen* OR youth* OR teen* OR schoolchild* OR "school-aged" OR pediatric* OR paediatric*)

Digital food-literacy/education (D)

("digital food literacy" OR "digital nutrition literacy"

OR ((digital OR online OR "web-based" OR mobile OR app* OR smartphone*

OR game* OR gamif* OR "social media" OR "virtual reality"

OR "augmented reality" OR chatbot* OR "digital platform*")

NEAR/3

("food literacy" OR "nutrition literacy" OR "food education"

OR "nutrition education" OR "food system* education" OR "media literacy")))

Scopus used `W/3` in place of `NEAR/3`.

Sustainability (S)

("sustainable diet*" OR "sustainable eating" OR "sustainable food choice*"

OR "healthy and sustainable diet*" OR "planetary health diet*"

OR "climate-friendly diet*" OR "plant-based diet*" OR "food waste"

OR "food system*" OR "environmental sustainability" OR "responsible consumption")

Education/action (E)

(educat* OR intervention* OR program* OR curriculum* OR nudge* OR literacy OR learn*)

## 1.4 Web of Science Core Collection

Field: Topic. Database version: all editions available through the institutional session.

Strict branch

TS=(D AND S AND P)

The interface query was:

(("digital food literacy" OR "digital nutrition literacy"

OR ((digital OR online OR "web-based" OR mobile OR app* OR smartphone*

OR game* OR gamif* OR "social media" OR "virtual reality"

OR "augmented reality" OR chatbot* OR "digital platform*")

NEAR/3

("food literacy" OR "nutrition literacy" OR "food education"

OR "nutrition education" OR "food system* education" OR "media literacy")))

AND

("sustainable diet*" OR "sustainable eating" OR "sustainable food choice*"

OR "healthy and sustainable diet*" OR "planetary health diet*"

OR "climate-friendly diet*" OR "plant-based diet*" OR "food waste"

OR "food system*" OR "environmental sustainability" OR "responsible consumption")

AND

(child* OR adolescen* OR youth* OR teen* OR schoolchild* OR "school-aged"

OR pediatric* OR paediatric*))

Raw result: 18. No interface date, language, or document-type filters were applied. Full records and cited references were exported. Eligibility filters were applied during screening, which transparently removed a 1998 article and a proceedings paper.

Calibration branches

TS=(D AND P)	-> 636 records

TS=(S AND E AND P)	-> 1,556 records

These counts were not exported or screened.

## 1.5 Scopus

Field: `TITLE-ABS-KEY`.

Strict branch

TITLE-ABS-KEY(

("digital food literacy" OR "digital nutrition literacy"

OR ((digital OR online OR "web-based" OR mobile OR app* OR smartphone*

OR game* OR gamif* OR "social media" OR "virtual reality"

OR "augmented reality" OR chatbot* OR "digital platform*")

W/3

("food literacy" OR "nutrition literacy" OR "food education"

OR "nutrition education" OR "food system* education" OR "media literacy")))

AND

("sustainable diet*" OR "sustainable eating" OR "sustainable food choice*"

OR "healthy and sustainable diet*" OR "planetary health diet*"

OR "climate-friendly diet*" OR "plant-based diet*" OR "food waste"

OR "food system*" OR "environmental sustainability" OR "responsible consumption")

AND

(child* OR adolescen* OR youth* OR teen* OR schoolchild* OR "school-aged"

OR pediatric* OR paediatric*)

)

AND PUBYEAR > 1999 AND PUBYEAR < 2027

AND (DOCTYPE(ar) OR DOCTYPE(re)) AND SRCTYPE(j) AND LANGUAGE(english)

Raw result: 15 records (14 articles, 1 review). All records and all available fields were exported to CSV.

Calibration branches

The shorthand below is a human-readable audit notation, not a second set of database-ready strings: `D`, `S`, `E`, and `P` refer to the complete term blocks printed in the strict Scopus query above, and `D-with-W/3` applies `W/3` between the digital-medium terms and the intervention/education terms. These branches were used only to estimate neighboring-field size and were not screened or included in the review corpus.

TITLE-ABS-KEY(D-with-W/3 AND P)

AND PUBYEAR > 1999 AND PUBYEAR < 2027

AND (DOCTYPE(ar) OR DOCTYPE(re)) AND SRCTYPE(j) AND LANGUAGE(english)

-> 943 records

TITLE-ABS-KEY(S AND E AND P)

AND PUBYEAR > 1999 AND PUBYEAR < 2027

AND (DOCTYPE(ar) OR DOCTYPE(re)) AND SRCTYPE(j) AND LANGUAGE(english)

-> 1,642 records

The adjacent results were used only to demonstrate that each neighboring literature is large while their direct overlap is sparse.

## 1.6 PubMed

The following query is executable as written:

((

"digital food literacy"[tiab] OR "digital nutrition literacy"[tiab]

OR (

(digital[tiab] OR online[tiab] OR "web-based"[tiab] OR mobile[tiab]

OR app[tiab] OR apps[tiab] OR smartphone*[tiab] OR game*[tiab]

OR gamif*[tiab] OR "social media"[tiab] OR "virtual reality"[tiab]

OR "augmented reality"[tiab] OR chatbot*[tiab]

OR "digital platform"[tiab] OR "digital platforms"[tiab])

AND

("food literacy"[tiab] OR "nutrition literacy"[tiab]

OR "food education"[tiab] OR "nutrition education"[tiab]

OR "food systems education"[tiab] OR "media literacy"[tiab])

)

)

AND

("sustainable diet"[tiab] OR "sustainable diets"[tiab]

OR "sustainable eating"[tiab] OR "sustainable food choice"[tiab]

OR "sustainable food choices"[tiab]

OR "healthy and sustainable diet"[tiab]

OR "healthy and sustainable diets"[tiab]

OR "planetary health diet"[tiab] OR "climate-friendly diet"[tiab]

OR "plant-based diet"[tiab] OR "plant-based diets"[tiab]

OR "food waste"[tiab] OR "food system"[tiab] OR "food systems"[tiab]

OR "environmental sustainability"[tiab]

OR "responsible consumption"[tiab])

AND

(child*[tiab] OR adolescen*[tiab] OR youth*[tiab] OR teen*[tiab]

OR schoolchild*[tiab] OR "school-aged"[tiab]

OR pediatric*[tiab] OR paediatric*[tiab])

)

AND english[lang]

AND ("2000/01/01"[dp] : "2026/12/31"[dp])

AND (journal article[pt] OR review[pt])

Raw result: 15 PMIDs. PubMed reported that the exact quoted phrases `"digital food literacy"` and `"climate-friendly diet"` were not in its phrase index; the remaining Boolean components were translated and executed. This warning is retained because it affects reproducibility.

## 1.7 ERIC

Endpoint: `[https://api.ies.ed.gov/eric/`](https://api.ies.ed.gov/eric/%60).

The API request used `format=json`, `rows=200`, and

`fields=id,title,author,description,subject,publicationtype,publicationdateyear,language,issn,publisher,peerreviewed`.

The complete Boolean expression below was passed as the `search` parameter.

(("digital food literacy" OR "digital nutrition literacy"

OR ((digital OR online OR mobile OR smartphone OR game OR gamification

OR "social media" OR "virtual reality" OR chatbot)

AND

("food literacy" OR "nutrition literacy" OR "food education"

OR "nutrition education" OR "food systems education" OR "media literacy")))

AND

("sustainable diet" OR "sustainable eating" OR "sustainable food choice"

OR "planetary health diet" OR "plant-based diet" OR "food waste"

OR "food system" OR "environmental sustainability"

OR "responsible consumption")

AND

(child OR children OR adolescent OR adolescents OR youth OR teen

OR schoolchild OR "school-aged"))

AND peerreviewed:T

AND publicationdateyear:[2000 TO 2026]

AND language:English

Raw result: 5 records. Because ERIC's search syntax is less field-specific, records were screened conservatively; for example, a youth/TikTok sustainability perspective with no food construct was excluded.

## 1.8 Deduplication and screening

The four strict exports contained 53 database records. A scripted normalization procedure normalized DOI strings and then used a conservative normalized-title fallback, producing 38 unique database candidates. Citation checking and updated closest-review screening recovered one additional eligible intervention, producing 39 screened candidates. Every candidate was coded against the operational P1, D1, L1, S1, I1, DOC1, and DATE1 criteria. Three empirical studies met all strict-core criteria. Full-text checking also positioned an alternate-reality game as adjacent sustainability evidence because the intervention used game mechanics without a digital medium. Records meeting the digital-food-literacy side or the sustainability-food-education side were retained in mutually exclusive adjacent buckets.

The exact decisions, failure codes, and reasons are recorded in Supplementary Table S2; the supplemental route is recorded in the supplementary-candidate provenance statement above.

# 2 Appendix S1B. Expanded sensitivity searches

## 2.1 Expanded sensitivity searches run 20 August 2026

These exploratory high-sensitivity checks tested whether broader labels for digital mechanisms and sustainability would recover additional plausible candidates. Counts are non-additive and are not part of the original 39-record flow. No updated cross-database deduplicated corpus or second formal screening flow was constructed.

## 2.2 Web of Science Core Collection

Field: Topic. Raw count: 154.

(("digital food literacy" OR "digital nutrition literacy" OR ((digital OR online OR "web-based" OR "internet-based" OR mobile OR mHealth OR eHealth OR app* OR smartphone* OR SMS OR "text messag*" OR multimedia OR video* OR game* OR gamif* OR "serious game*" OR simulation* OR "virtual reality" OR "augmented reality" OR remote OR "social media" OR "digital platform*") NEAR/5 ("food literacy" OR "nutrition literacy" OR "food education" OR "nutrition education" OR "food skill*" OR "cooking skill*" OR "meal planning" OR "food competenc*" OR cook*)) OR (((digital OR online OR "web-based" OR "internet-based" OR mobile OR mHealth OR eHealth OR app* OR smartphone* OR SMS OR "text messag*" OR multimedia OR video* OR game* OR gamif* OR "serious game*" OR simulation* OR "virtual reality" OR "augmented reality" OR remote OR "social media" OR "digital platform*") NEAR/5 ("sustainable diet*" OR "sustainable eating" OR "plant-based diet*" OR "plant-forward" OR "meat reduction" OR "food waste" OR "food loss" OR "food system*" OR "environmental sustainability" OR "carbon footprint*")) AND (educat* OR intervention* OR program* OR curricul* OR learn* OR literacy OR skill*))) AND ("sustainable diet*" OR "sustainable eating" OR "sustainable food choice*" OR "healthy and sustainable diet*" OR "planetary health diet*" OR "climate-friendly diet*" OR "low-carbon diet*" OR "plant-based diet*" OR "plant-forward" OR "meat reduction" OR "food waste" OR "food loss" OR "food system*" OR "environmental sustainability" OR "dietary carbon" OR "carbon footprint*" OR "responsible consumption") AND (child* OR adolescen* OR youth* OR teen* OR schoolchild* OR "school-aged" OR pediatric* OR paediatric*))

## 2.3 Scopus

Field: TITLE-ABS-KEY. Raw count: 184. No interface filters were applied to the raw count.

TITLE-ABS-KEY((("digital food literacy" OR "digital nutrition literacy" OR ((digital OR online OR "web-based" OR "internet-based" OR mobile OR mHealth OR eHealth OR app* OR smartphone* OR SMS OR "text messag*" OR multimedia OR video* OR game* OR gamif* OR "serious game*" OR simulation* OR "virtual reality" OR "augmented reality" OR remote OR "social media" OR "digital platform*") W/5 ("food literacy" OR "nutrition literacy" OR "food education" OR "nutrition education" OR "food skill*" OR "cooking skill*" OR "meal planning" OR "food competenc*" OR cook*)) OR (((digital OR online OR "web-based" OR "internet-based" OR mobile OR mHealth OR eHealth OR app* OR smartphone* OR SMS OR "text messag*" OR multimedia OR video* OR game* OR gamif* OR "serious game*" OR simulation* OR "virtual reality" OR "augmented reality" OR remote OR "social media" OR "digital platform*") W/5 ("sustainable diet*" OR "sustainable eating" OR "plant-based diet*" OR "plant-forward" OR "meat reduction" OR "food waste" OR "food loss" OR "food system*" OR "environmental sustainability" OR "carbon footprint*")) AND (educat* OR intervention* OR program* OR curricul* OR learn* OR literacy OR skill*))) AND ("sustainable diet*" OR "sustainable eating" OR "sustainable food choice*" OR "healthy and sustainable diet*" OR "planetary health diet*" OR "climate-friendly diet*" OR "low-carbon diet*" OR "plant-based diet*" OR "plant-forward" OR "meat reduction" OR "food waste" OR "food loss" OR "food system*" OR "environmental sustainability" OR "dietary carbon" OR "carbon footprint*" OR "responsible consumption") AND (child* OR adolescen* OR youth* OR teen* OR schoolchild* OR "school-aged" OR pediatric* OR paediatric*)))

## 2.4 PubMed

Raw count: 96.

(("digital food literacy"[tiab] OR "digital nutrition literacy"[tiab] OR (((digital[tiab] OR online[tiab] OR "web-based"[tiab] OR "internet-based"[tiab] OR mobile[tiab] OR mHealth[tiab] OR eHealth[tiab] OR app*[tiab] OR smartphone*[tiab] OR SMS[tiab] OR "text message"[tiab] OR "text messages"[tiab] OR multimedia[tiab] OR video*[tiab] OR game*[tiab] OR gamif*[tiab] OR "serious game"[tiab] OR "serious games"[tiab] OR simulation*[tiab] OR "virtual reality"[tiab] OR "augmented reality"[tiab] OR remote[tiab] OR "social media"[tiab] OR "digital platform"[tiab] OR "digital platforms"[tiab]) AND ("food literacy"[tiab] OR "nutrition literacy"[tiab] OR "food education"[tiab] OR "nutrition education"[tiab] OR "food skill"[tiab] OR "food skills"[tiab] OR "cooking skill"[tiab] OR "cooking skills"[tiab] OR "meal planning"[tiab] OR "food competence"[tiab] OR cook*[tiab])) OR (((digital[tiab] OR online[tiab] OR "web-based"[tiab] OR "internet-based"[tiab] OR mobile[tiab] OR mHealth[tiab] OR eHealth[tiab] OR app*[tiab] OR smartphone*[tiab] OR SMS[tiab] OR multimedia[tiab] OR video*[tiab] OR game*[tiab] OR gamif*[tiab] OR simulation*[tiab] OR remote[tiab] OR "social media"[tiab]) AND ("sustainable diet"[tiab] OR "sustainable eating"[tiab] OR "plant-based diet"[tiab] OR "plant-forward"[tiab] OR "meat reduction"[tiab] OR "food waste"[tiab] OR "food loss"[tiab] OR "food system"[tiab] OR "food systems"[tiab] OR "environmental sustainability"[tiab] OR "carbon footprint"[tiab])) AND (educat*[tiab] OR intervention*[tiab] OR program*[tiab] OR curricul*[tiab] OR learn*[tiab] OR literacy[tiab] OR skill*[tiab]))) AND ("sustainable diet"[tiab] OR "sustainable diets"[tiab] OR "sustainable eating"[tiab] OR "sustainable food choice"[tiab] OR "healthy and sustainable diet"[tiab] OR "planetary health diet"[tiab] OR "low-carbon diet"[tiab] OR "plant-based diet"[tiab] OR "plant-forward"[tiab] OR "meat reduction"[tiab] OR "food waste"[tiab] OR "food loss"[tiab] OR "food system"[tiab] OR "food systems"[tiab] OR "environmental sustainability"[tiab] OR "dietary carbon"[tiab] OR "carbon footprint"[tiab] OR "responsible consumption"[tiab]) AND (child*[tiab] OR adolescen*[tiab] OR youth*[tiab] OR teen*[tiab] OR schoolchild*[tiab] OR "school-aged"[tiab] OR pediatric*[tiab] OR paediatric*[tiab]))) AND english[lang] AND ("2000/01/01"[dp] : "2026/08/20"[dp]) AND (journal article[pt] OR review[pt])

PubMed warned that `"digital food literacy"[tiab]` and `"food competence"[tiab]` were not in its phrase index. The Boolean translation and warnings are retained in the audit log.

## 2.5 ERIC

Endpoint: `[https://api.ies.ed.gov/eric/`](https://api.ies.ed.gov/eric/%60). Raw count: 35. Parameters: `format=json`, `rows=200`, `fields=id,title,author,description,subject,publicationtype,publicationdateyear,language,issn,publisher,peerreviewed`.

((digital OR online OR mobile OR smartphone OR game OR gamification OR "social media" OR "virtual reality" OR simulation OR video OR multimedia) AND (food OR nutrition OR cooking) AND (education OR learning OR literacy OR intervention OR program) AND (sustainability OR "food waste" OR "food system" OR "sustainable diet") AND (child OR children OR adolescent OR adolescents OR youth OR teen OR school)) AND peerreviewed:T AND publicationdateyear:[2000 TO 2026] AND language:English

## 2.6 Exploratory sensitivity-check outcome

Title and metadata inspection in Scopus, PubMed, and ERIC, supplemented by Web of Science interface checks, identified no evident additional record meeting P1, D1, L1, S1, and I1 among the records examined. Important near-matches included a 2026 cross-sectional study of social-media use and adolescent dietary carbon footprints (no delivered education), an alternate-reality game that explicitly used no digital device (D0), young-adult digital sustainable-nutrition programs (P0), and conference or prototype reports without an eligible journal intervention evaluation (DOC0/I0). These exploratory checks test vocabulary sensitivity and are not reported as a completed updated study flow.

Table S2. Screening decisions, failed gates, and reasons

| **ID** | **Record** | **Decision** | **Full text?** | **Failed gate(s)** | **Reason** |
| --- | --- | --- | --- | --- | --- |
| C001 | Food systems and market pathways to childhood stunting in mining communities: evidence from a cross-sectional survey and a synthesis of context-responsive nutrition interventions. | Excluded | No | P0;D0;L0;I0 | Under-five/caregiver cross-sectional study; digital platforms appear only as a recommendation, not a delivered intervention. |
| C002 | The Association Between Social Media Usage on Food Choice Motivations and Dietary Carbon Footprints in Adolescents: A Cross-Sectional Study | Context record | Yes | L0;I0 | Directly links adolescents' social-media environment to food-choice motives and dietary carbon footprint, but it is observational and does not deliver food-literacy education; it is context rather than adjacent digital food-literacy evidence. |
| C003 | The LO-VEg Project—A School-Based Nudging and Communication Intervention to Promote Vegetable and Legume Consumption: Preliminary Evidence from an Ecological Study in Italian Primary Schools. | Adjacent boundary record | Yes | D0 | The broader architecture included digital components, but the published evaluation explicitly focuses on school-based nudging/communication and does not evaluate a substantive digital educational component. |
| C004 | Children and teens must develop digital literacy skills to navigate complex and evolving food ecosystems | Context record | Yes | I0;DOC0 | Direct conceptual bridge among digital food/nutrition literacy, young people, dietary choice, and sustainable food systems; a letter, not intervention evidence. |
| C005 | Digital interventions in nutrition education: a web-based model for promoting sustainable nutrition among young adults | Excluded | No | P0 | Eligible concept and intervention but sample is young adults rather than a separable 5–18-year population. |
| C006 | Evaluation of the Influence of Intervention Tools Used in Nutrition Education Programs: A Mixed Approach | Excluded | No | D0;S0;I0 | Review concerns school nutrition education and Mediterranean-diet adherence; it neither isolates digital strategies nor operationalizes sustainability. |
| C007 | Food literacy in preschool-aged children—Influencing factors, programmes, and outcomes: A scoping review | Excluded | No | P0;D0;S0;I0 | Preschool scope falls below the selected age boundary and review does not center digital delivery or sustainability. |
| C008 | Opportunities for enhancing dietary diversity and environmental sustainability of supplementary nutrition program under the Integrated Child Development Services Scheme—a qualitative study in Srikakulam district, Andhra Pradesh. | Excluded | No | P0;D0;L0;I0 | Stakeholder/implementation study of an under-five public nutrition program; online application is an operational barrier, not an educational component. |
| C009 | Promoting Learning About Nutrition and Healthy Eating Behaviors in Chinese Children Through an Alternate Reality Game: A Pilot Study. | Adjacent boundary record | Yes | D0 | The game delivered nutrition tasks and directly weighed food waste, but the full text explicitly states that HFHM operated without any digital devices and was presented as an alternative to digital serious games. |
| C010 | Shaping Sustainable Futures on TikTok? Media Literacy, Youth Education and the SDGs | Excluded | No | L0;I0 | Relevant platform/media-literacy perspective but contains no food or nutrition construct. |
| C011 | The Whole School Food Approach: A European framework and implementation to promote healthy and sustainable school food systems. | Adjacent boundary record | No | D0 | Whole-school sustainable food framework; online surveys were data collection rather than digital pedagogy. |
| C012 | Vegetable consumption and promotion among school-age children and adolescents in West Africa: a systematic review and narrative synthesis. | Excluded | No | D0;S0;I0 | Vegetable-promotion review; sustainability is not an explicit intervention construct and digital delivery is not synthesized. |
| C013 | ARFood: Pioneering Nutrition Education for Generation Alpha Through Augmented Reality and AI-Driven Serious Gaming | Excluded | No | DOC0 | Highly relevant AR/AI prototype, but indexed as a proceedings paper and the exported abstract does not provide an auditable evaluation design or results. |
| C014 | Comparison of Food Selection with the National School Lunch Program Meal Pattern Guidelines and Assessment of Children's Food Intake and Waste Using Digital Photography in a School Cafeteria. | Excluded | No | D0;L0;I0 | Digital photography was used only to measure intake and waste; no educational intervention was delivered. |
| C015 | Enhancing Food and Nutrition Literacy: A Key Strategy for Reducing Food Waste and Improving Diet Quality | Excluded | No | P0;D0;I0 | General consumer commentary/review with no child/adolescent population and no digital intervention. |
| C016 | Food, nutrition and sustainability education in Australian primary schools: a cross-sectional analysis of teacher perspectives and practices. | Adjacent boundary record | Yes | D0;I0 | Maps delivery barriers and teacher capacity for sustainability education; online survey is data collection only. |
| C017 | Household Food Waste Intervention Is Feasible, Acceptable, and Effective. | Direct inclusion | Yes | — | Whole-family food-literacy intervention used an online cooking class and repeated text messages to build meal-preparation and waste-reduction skills; pre/post waste audits measured material food-waste change. |
| C018 | Impact and process evaluation of a primary-school Food Education and Sustainability Training (FEAST) program in 10–12-year-old children in Australia: pragmatic cluster non-randomized controlled trial. | Adjacent boundary record | Yes | D0 | Sustainability/food-literacy curriculum; partial online delivery occurred because of school closures and was not a designed digital-literacy mechanism. |
| C019 | Striving for a holistic approach: exploring food education through Finnish youth centers | Adjacent boundary record | No | D0 | Youth food-education study explicitly addresses food systems/environment but has no digital educational component. |
| C020 | 'Hip-Hop & Food': A participatory approach to informal nutrition education in the youth center setting within the IN FORM project KlimaFood | Adjacent boundary record | No | D0 | Climate-friendly youth nutrition workshop; hip-hop is a participatory cultural method, not a digital platform. |
| C021 | Evidence-Based Design Process for Nutrition-Sensitive Agriculture Interventions: A Case Study of the Advancing Local Dairy Development Programme in Nigeria | Excluded | No | P0;D0;L0;I0 | Household/adult agriculture-program design study; children under five are beneficiaries rather than educational participants. |
| C022 | Rethinking Adolescent School Nutrition Education Through a Food Systems Lens | Direct inclusion | Yes | — | Purpose-built computer game simulated food supply chains and resource waste, followed by structured inquiry with 13–16-year-olds. |
| C023 | Understanding school food systems to support the development and implementation of food based policies and interventions | Excluded | No | D0;L0;I0 | Participatory system-mapping study of school food environments; no digital food-literacy strategy was delivered. |
| C024 | COVID-19 and the National Lockdown: How Food Choice and Dietary Habits Changed for Families in the United Kingdom | Excluded | No | D0;L0;I0 | Observational family interview study; pandemic context and online-era behavior do not constitute a digital intervention. |
| C025 | Developing culturally appropriate food literacy resources for Aboriginal children with Foodbank WA’s Superhero Foods® | Context record | Yes | S0 | Culturally adapted resources had free online access and support equity/food sovereignty, but the evaluated outcomes were healthy-eating/cultural relevance rather than explicit sustainable-diet outcomes. |
| C026 | Essential Food and Nutrition Knowledge and Skills for Primary School Children: Australian Parents' Opinions | Excluded | No | D0;L0;I0 | Parent online survey about curriculum priorities; no digital child education was delivered. |
| C027 | How Primary School Curriculums in 11 Countries around the World Deliver Food Education and Address Food Literacy: A Policy Analysis | Adjacent boundary record | No | D0;I0 | Useful policy analysis showing weak curriculum coverage of sustainability/equity, but no digital pedagogy or intervention evaluation. |
| C028 | Evaluating OzHarvest's primary-school Food Education and Sustainability Training (FEAST) program in 10–12-year-old children in Australia: protocol for a pragmatic cluster non-randomized controlled trial. | Excluded | Yes | D0;I0 | Protocol only; outcome evidence is represented by the completed FEAST evaluation (C018). |
| C029 | FOODLIT-PRO: conceptual and empirical development of the food literacy wheel | Excluded | No | P0;D0;I0 | Expert-derived adult/general conceptual framework, with no child digital intervention. |
| C030 | How the collaborative work of farm to school can disrupt neoliberalism in public schools | Adjacent boundary record | No | D0 | School food-systems education and equity case study without a digital component. |
| C031 | Healthy Planet, Healthy Youth: A Food Systems Education and Promotion Intervention to Improve Adolescent Diet Quality and Reduce Food Waste. | Adjacent boundary record | Yes | D0 | Direct sustainability education with behavioral/plate-waste outcomes; digital photography was outcome measurement only. |
| C032 | Understanding Food Literacy in Urban Gardeners: A Case Study of the Twin Cities, Minnesota | Excluded | No | P0;D0;I0 | Adult urban-gardener survey, not a child digital intervention. |
| C033 | Unravelling the food literacy puzzle: Evidence from Italy | Excluded | No | P0;D0;I0 | General adult population food-literacy measurement study. |
| C034 | Food Consumers' Views of Essential Food Knowledge and Skills for All Consumers | Excluded | No | P0;D0;L0;I0 | Adult online surveys; reference to secondary-school food education is contextual only. |
| C035 | A mobile farmers’ market brings nutrition education to low-income students | Adjacent boundary record | No | D0 | Mobile refers to a converted bus, not digital delivery; provides hands-on sustainable-farming education. |
| C036 | Food-System Botany | Adjacent boundary record | No | D0 | Inquiry lesson set supports food-system literacy but online resources are supplementary rather than a digital pedagogy. |
| C037 | Nutrition education and support program for community-dwelling adults with intellectual disabilities | Excluded | No | P0;D0;S0 | Adult group-home intervention; child indexing is spurious. |
| C038 | The cookshop program: Outcome evaluation of a nutrition education program linking lunchroom food experiences with classroom cooking experiences | Excluded | No | D0;DATE0 | Relevant historical sustainability/cooking intervention but predates the formal 2000 lower bound and has no digital component. |
| C039 | Feasibility, acceptability, and preliminary impact of “Supper Heroes”, a family-based sustainable diet intervention | Direct inclusion | Yes | — | Four-month family mHealth intervention delivered multimedia sustainable-diet education and activities, with food-waste, plant-protein, and child food-literacy outcomes. |

Note. Decisions are mutually exclusive. Full-text-assessed records comprise three direct inclusions, five records without substantive digital pedagogy, three observational/conceptual/protocol reports, and one record without an explicit sustainability target in evaluated content or outcomes.

Table S3. Item-level MMAT 2018 appraisal

| **Study** | **Design component** | **MMAT item** | **Judgment** | **Rationale** |
| --- | --- | --- | --- | --- |
| Miller et al. (2023) | Screening | S1 clear research questions | Yes | The study states a focused qualitative question about educational content and pedagogy after game play. |
| Miller et al. (2023) | Screening | S2 data address research questions | Yes | Facilitated group-discussion data directly address learning and interests. |
| Miller et al. (2023) | Qualitative | 1.1 appropriate qualitative approach | Yes | A qualitative case study fits exploration of student learning and interests. |
| Miller et al. (2023) | Qualitative | 1.2 adequate qualitative data collection | Yes | Facilitated group discussions across five schools elicited relevant accounts. |
| Miller et al. (2023) | Qualitative | 1.3 findings derived from data | Yes | Reported themes are linked to student discussion data. |
| Miller et al. (2023) | Qualitative | 1.4 interpretation substantiated by data | Yes | Interpretations are supported by themes and illustrative accounts. |
| Miller et al. (2023) | Qualitative | 1.5 coherence | Yes | Question, data collection, analysis, and interpretation are coherent; transfer remains game-specific and immediate. |
| Laila et al. (2024) | Screening | S1 clear research questions | Yes | Feasibility, acceptability, and preliminary food-waste outcomes are stated. |
| Laila et al. (2024) | Screening | S2 data address research questions | Yes | Attendance, satisfaction, and household-waste data address the stated objectives. |
| Laila et al. (2024) | Quantitative non-randomized | 3.1 representative participants | No | A small volunteer convenience sample of 19 families does not establish representativeness. |
| Laila et al. (2024) | Quantitative non-randomized | 3.2 appropriate measurements | Yes | Program completion, satisfaction, and household-waste audits align with feasibility and outcome questions. |
| Laila et al. (2024) | Quantitative non-randomized | 3.3 complete outcome data | Unclear | Denominators differ across baseline, class attendance, parent surveys, child surveys, and waste outcomes. |
| Laila et al. (2024) | Quantitative non-randomized | 3.4 confounders accounted for | No | The single-arm pre–post design cannot separate intervention change from secular change or measurement effects. |
| Laila et al. (2024) | Quantitative non-randomized | 3.5 intervention administered as intended | Unclear | Eighteen of 19 families attended the scheduled class, but delivery does not establish use of the toolkit or intended message dose. |
| Eckert et al. (2025) | Screening | S1 clear research questions | Yes | Feasibility, acceptability, and preliminary impact objectives are stated. |
| Eckert et al. (2025) | Screening | S2 data address research questions | Yes | Analytics, surveys, interviews, diet measures, literacy measures, and weighed diaries address the objectives. |
| Eckert et al. (2025) | Qualitative | 1.1 appropriate qualitative approach | Yes | Interviews are appropriate for acceptability and implementation experience. |
| Eckert et al. (2025) | Qualitative | 1.2 adequate qualitative data collection | Yes | In-depth parent interviews provide detailed implementation accounts. |
| Eckert et al. (2025) | Qualitative | 1.3 findings derived from data | Yes | Themes are derived from interview material. |
| Eckert et al. (2025) | Qualitative | 1.4 interpretation substantiated by data | Yes | Acceptability interpretations are supported by reported parent accounts. |
| Eckert et al. (2025) | Qualitative | 1.5 coherence | Yes | Qualitative questions, interviews, analysis, and interpretation are coherent. |
| Eckert et al. (2025) | Quantitative non-randomized | 3.1 representative participants | No | The volunteer sample was small, geographically limited, and highly educated. |
| Eckert et al. (2025) | Quantitative non-randomized | 3.2 appropriate measurements | Yes | Website analytics, defined questionnaires, diet measures, and weighed waste diaries align with the outcomes. |
| Eckert et al. (2025) | Quantitative non-randomized | 3.3 complete outcome data | No | Twenty-three of 30 families were retained and only 16 provided paired weighed diaries. |
| Eckert et al. (2025) | Quantitative non-randomized | 3.4 confounders accounted for | No | The single-arm pre–post design lacks a concurrent control and confounder adjustment for change. |
| Eckert et al. (2025) | Quantitative non-randomized | 3.5 intervention administered as intended | Unclear | Analytics documented exposure, but 19 of 25 starters completed all seven missions and no prespecified fidelity threshold was reported. |
| Eckert et al. (2025) | Mixed methods | 5.1 adequate rationale for mixed methods | Yes | Quantitative feasibility/impact measures and interviews address complementary implementation questions. |
| Eckert et al. (2025) | Mixed methods | 5.2 components effectively integrated | Yes | Survey, analytic, outcome, and interview findings are combined in the interpretation. |
| Eckert et al. (2025) | Mixed methods | 5.3 interpretation of integrated outputs | Yes | The discussion uses multiple data types to interpret feasibility and family participation. |
| Eckert et al. (2025) | Mixed methods | 5.4 divergences addressed | Unclear | Differences among parent satisfaction, child engagement, and outcome change are noted but not formally integrated as divergent findings. |
| Eckert et al. (2025) | Mixed methods | 5.5 components meet quality criteria | No | The quantitative component remains limited by single-arm design and incomplete outcome data. |

Note. MMAT guidance discourages a single numerical score; judgments are therefore presented item by item. No study was excluded on quality.

Table S4. Backward and forward citation checking

| **Seed report** | **Backward reference checking** | **Forward/update checking** | **Outcome** |
| --- | --- | --- | --- |
| Miller et al. (2023) | Publisher full reference list checked for earlier child/adolescent digital food-literacy interventions with explicit sustainability | Web of Science cited-by records and related-review included-study lists checked to 20 August 2026 | No additional eligible intervention; Miller remained a direct study |
| Laila et al. (2024) | Article reference list/cited-reference metadata checked for earlier child/family digital food-literacy interventions with explicit sustainability | Web of Science cited-by records and related-review included-study lists checked to 20 August 2026 | No additional eligible intervention; Laila remained a direct study |
| Eckert et al. (2025) | Author-manuscript reference list checked; Weeknight Supper Savers linked as a distinct earlier intervention | Web of Science cited-by records and 2026 digital sustainable-eating review checked to 20 August 2026 | No additional eligible intervention; Eckert remained a distinct direct study |
